# Emerging role of microglia in inter-cellular transmission of α-synuclein in Parkinson’s disease

**DOI:** 10.3389/fnagi.2024.1411104

**Published:** 2024-10-09

**Authors:** Xiangbo Zhang, Haiyang Yu, Juan Feng

**Affiliations:** Department of Neurology, Shengjing Hospital of China Medical University, Shenyang, China

**Keywords:** Parkinson’s disease, microglia, α-synuclein, neurodegeneration, propagation

## Abstract

Parkinson’s disease (PD) is the second most common neurodegenerative disease worldwide, significantly prejudicing the health and quality of life of elderly patients. The main pathological characteristics of PD are the loss of dopaminergic neurons in the substantia nigra (SN) as well as abnormal aggregation of α-synuclein (α-syn) monomers and oligomers, which results in formation of Lewy bodies (LBs). Intercellular transmission of α-syn is crucial for PD progression. Microglia play diverse roles in physiological and pathological conditions, exhibiting neuroprotective or neurotoxic effects; moreover, they may directly facilitate α-syn propagation. Various forms of extracellular α-syn can be taken up by microglia through multiple mechanisms, degraded or processed into more pathogenic forms, and eventually released into extracellular fluid or adjacent cells. This review discusses current literature regarding the molecular mechanisms underlying the uptake, degradation, and release of α-syn by microglia.

## 1 Introduction

Microglia are macrophages residing in the brain, originating from early erythroid myeloid progenitors in the embryo, which is much earlier than the development stage of other glial cells (Prinz et al., 2019). Under physiological and most pathological conditions, microglia are responsible for clearing dead neurons, pruning excessive synapses, and removing misfolded or aggregated proteins. Microglia express various proteins that respond to damage-associated molecular patterns, including Toll-like receptors (TLR) and Fc receptors, and trigger neuroinflammatory responses through cytokines such as interleukin (IL) -6, IL-1β, and tumor necrosis factor α (TNF-α) (Kann et al., 2022). On one hand, this inflammatory response can be limited to microglial proliferation and activation or recruitment of peripheral immune cells from the circulation system via the release of chemokines, such as C-C motif ligand 2 (CCL2) (Weiss et al., 1998), to facilitate the clearance of toxic substances and maintenance of the microenvironment. On the other hand, sustained release of cytokines and excessive neurotransmitters, such as glutamate, can cause neuronal damage. The role of microglia in neurodegenerative diseases, including multiple sclerosis, Alzheimer’s disease, amyotrophic lateral sclerosis, and PD, is often complex and bidirectional (Sarlus and Heneka, 2017; Muzio et al., 2021). TNF-α released by microglia in SN can cause neurodegeneration of dopaminergic cells in PD (De Lella Ezcurra et al., 2010), and concurrently, alternative activation of microglia can release anti-inflammatory cytokines such as IL-4 and IL-13.

Since the pioneering autopsy study on patients with PD conducted by Braak et al. (2003) reported abnormal α-syn aggregates in the autonomic nervous system (ANS) and the dorsal motor nucleus of the vagus (DMV), subsequent studies confirmed that both the sympathetic and parasympathetic nervous systems (especially the vagus nerve) could carry and transmit α-syn (Kim et al., 2019; Van Den Berge et al., 2019; Svensson et al., 2015). Although PD has an unclear pathogenesis, there has been increasing attention on the prion-like replication and propagation of α-syn between cells. α-syn aggregates are mainly generated in neurons. All α-syn configurations can be released into the extracellular space through the unconventional protein export pathway (Lee et al., 2005); degraded by various proteases, including cathepsin6 (Tatebe et al., 2010; Spencer et al., 2013) and plasmin (Kim et al., 2012); and internalized into neighboring neurons, astrocytes, and microglia (Hansen et al., 2011). Moreover, diffusion after neuronal death can expose these proteins to surrounding neurons and glial cells. Both astrocytes (Lee H. et al., 2010) and microglia can degrade α-syn produced by neurons. Under pathological conditions, microglia can act as transmission medium for α-syn, facilitating extensive effects. By injecting pre-formed fibrils (PFF) into the striatum of mice and observing distant regions, Kim et al. (2020) found that microglial activation was correlated with the spread and aggregation of phosphorylated α-syn, while the activation of astrocytes was only observed within the vicinity of the injection site. This demonstrated the correlation between microglia and the pathological spread of α-syn; however, the underlying mechanism remains unclear. This review aims to discuss the role of microglia in the uptake, degradation, and release of α-syn in PD.

## 2 Uptake of fibrils, soluble oligomers, and monomers of α-syn by microglia

### 2.1 Fibrils and oligomers

Both oligomeric and fibrillar forms of α-syn, including PFF, can enter cells through endocytosis *in vivo* as well as *in vitro*, with receptor-mediated endocytosis being a possible key mechanism (Reyes et al., 2014). Specific receptors on the cell membrane may directly or indirectly interact with oligomeric or fibrillar α-syn. Unlike monomeric α-syn, fibrillar α-syn may interact with the cell membrane surface of microglial cells and partially inhibit their phagocytic activity (Park et al., 2008). Several receptors for α-syn oligomers and fibrils have been reported.

#### 2.1.1 Toll-like receptor 2

**FIGURE 1:**
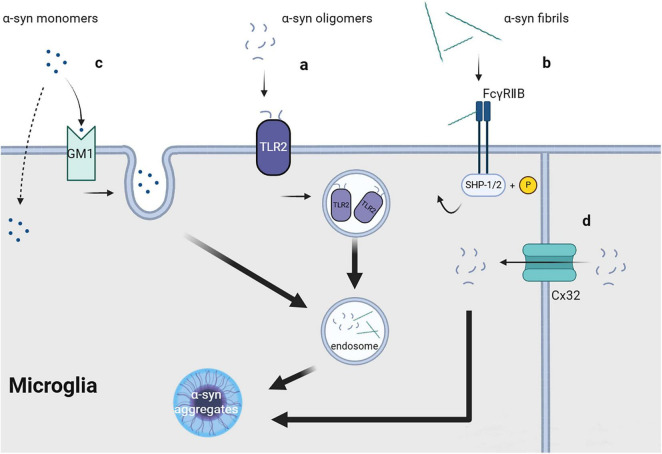
Uptake of fibrils, soluble oligomers and monomers of α-synuclein.

Toll-like receptor 2 (TLR2), which is a transmembrane protein primarily expressed in immune cells, epithelial cells, neurons, and glial cells (Rietdijk et al., 2016), can form dimers with TLR1 or TLR6 and function as a receptor recognizing exogenous and endogenous ligands, including bacterial metabolites (Akira and Takeda, 2004) and α-syn. Depending on the cell type, ligand, and co-receptor, TLR2 activation may yield pro- or anti-inflammatory responses. In microglia, TLR2 activation by aggregated β-amyloid 42 (Aβ42) can induce the release of pro-inflammatory cytokines, which can be enhanced by the co-receptor TLR1 or inhibited by TLR2-TLR6 activation (van Bergenhenegouwen et al., 2013). Oligomeric α-syn rich in β-sheet can directly interact with TLR2, with this interaction being conformation-sensitive, and may be internalized together with TLR2 (Figure 1a; Kim et al., 2013). TLR2 activation in neurons and astrocytes may inhibit α-syn clearance through the AKT-mTOR signaling pathway (Kim et al., 2015), which leads to increased α-syn aggregation. Contrastingly, TLR2 activation in microglia induces neurotoxic microglial activation through the NFκB and p38 MAPK signaling cascade (Choi et al., 2015), resulting in the production of neurotoxic substances, including cytokines, reactive oxygen species, and nitric oxide (Kim C. et al., 2018). Furthermore, another study strongly demonstrated that TLR2 can directly bind to exosomal α-syn as a receptor and promote microglial internalization of α-syn in general, which induces the pathological features of PD both *in vitro* and *in vivo* (Xia et al., 2021).

#### 2.1.2 Connexin

Connexins (Cx) are a series of transmembrane proteins and important components of gap junctions between cells. Gap junctions comprise two half channels connecting two cells, with each half channel comprising an assembly of six transmembrane Cx molecules, which have different functions depending on the types of Cx involved. Under physiological conditions, gap junctions are crucially involved in maintaining and promoting cellular homeostasis, allowing ions and molecules with a size <1 kDa (including sugars, amino acids, and nucleotides) to directly transfer between connected cells (Charvériat et al., 2021). In neurons and oligodendrocytes, Cx32 can bind to oligomeric α-syn and mediate the selective uptake of oligomeric α-syn, as well as monomeric and fibrillar α-syn, albeit to a lesser quantity (Figure 1d; Reyes et al., 2019). Cx32 may be expressed in microglia at the edge of brain tissue with traumatic injury; however, its specific function remains unclear (Moon et al., 2010).

#### 2.1.3 FcγRIIB

FcγRIIB is a cell surface inhibitory receptor that can interact with immune complexes (IC) at physiological levels and is widely expressed in hematopoietic cells. FcγRIIB can recruit the SH2 domain containing phosphatidylinositol 5′- phosphatase (SHIP) in cells by interacting with Aβ. This interferes with phosphoinositide metabolism, which aggravates memory impairment in patients with Alzheimer’s disease (Kam et al., 2016). Similar to Aβ, α-syn fibrils can directly interact with FcγRIIB on the surface of microglia or neurons and trigger raft-dependent internalization mediated by downstream SHP-1/SHP-2 phosphorylation (Figure 1b); however, monomeric α-syn lacks the same effect (Choi et al., 2015; Choi et al., 2018). The specific receptor for α-syn internalization in this process remains unclear.

#### 2.1.4 Heparan sulfate proteoglycan

Heparan sulfate proteoglycans (HSPG), which represent a class of glycoprotein that widely exists in cell membranes and extracellular matrix, are composed of different core proteins bound to heparan sulfate (HS) chains that contain repeating disaccharide units. The Syndecan family is the only core protein member in transmembrane HSPG. Specifically, its transmembrane domain induces the oligomerization of proteins that interact with its cytoplasmic domain, including β-amyloid protein and α-syn (Letoha et al., 2019). Syndecan-3 is commonly found in neurons, syndecan-4 is ubiquitous in various tissue systems (Bernfield et al., 1992; Afratis et al., 2017), and syndecan-2 is confirmed to express in ameboid microglial cells, macrophages in developing brain, while there is currently no report on mature microglia in adult central nervous system (CNS) (Kaur et al., 2009). Studies have shown that HSPG can specifically interact with α-syn fibril and mediate their intercellular propagation in neurons. Two different possible pathways, i.e., the raft-dependent and myosin-7B (MYO7B)-dependent pathways, have been recently reported. The C-terminal structure of MYO7B can bind to the cell membrane, promote the formation of “Ω-shaped” clathrin pits, and therefore enhances PFF internalization (Zhang et al., 2020). Other studies have demonstrated that PFF can be taken into the intracellular membrane system through endocytosis mediated by lipid rafts following interaction with HSPG (Hudák et al., 2019). Differently, heparin lyase treatment only slightly reduced fibril uptake in BV-2 cells, which suggest that microglia do not use HSPG as a major medium in α-syn internalization (Ihse et al., 2017). Given that immortalized cell lines may have deviation in physiological mechanism from original cells, more research is required in primary cells or *in vivo.*

#### 2.1.5 Prion protein

The prion protein (PrP*^C^*) has been reported to bind to α-syn fibrils and mediate their internalization in both *in vitro* and *in vivo* settings (Aulić et al., 2017). Although that microglia do not express PrP*^C^* and do not replicate scrapie prions (PrP*^Sc^*), it is reported that microglia colocalize with PrP*^Sc^* in mouses inoculated with hyposialylated PrP*^Sc^* (Makarava et al., 2020) suggesting that specially modified PrP*^C^* may recruit microglia and be phagocytosed by them through inflammatory response. This may be a different route of α-syn administration, However, another study reported contrasting results that PrP*^C^* neither bind α-syn oligomers nor participate in its neuro toxicity (La Vitola et al., 2019). Further research is required.

### 2.2 Monomers

Monomeric α-syn can be unselectively internalized by different cells, including neurons, astrocytes, microglia, and oligodendrocytes. The mechanism underlying the cellular entry of α-syn monomers; specifically, whether it involves direct transmembrane diffusion or receptor-mediated endocytosis, remains controversial. Some researchers believe that monomeric α-syn can directly penetrate the cell membrane, resulting in its non-selective cellular uptake (Lee, 2008; Lee H. J. et al., 2008). This is strongly demonstrated by the fact that the 11-amino acid imperfect repeat sequences of α-syn can easily pass cell membrane, which cannot be blocked by endocytic inhibitors or low temperature (Ahn et al., 2006). Contrastingly, other studies have suggested that the transmembrane process of monomeric α-syn may involve endocytic pathways. Monomeric α-syn uptake by microglia requires lipid raft-dependent endocytic pathways mediated by ganglioside GM1 and an unknown protein receptor (Figure 1c; Park et al., 2009). Other studies have reported that the cellular uptake of monomeric α-syn, both *in vivo* and *in vitro*, depends on dynamin (Reyes et al., 2014; Konno et al., 2012), which further supports the role of endocytosis in mediating the uptake of monomeric α-syn. This inconsistent reported could be attributed to variations in the purification conditions of monomeric α-syn. Additionally, whether these mechanisms are equally effective in microglia has not been directly confirmed, further research is required.

## 3 Intracellular clearance and degradation failure of α-syn by microglia

Microglia are responsible for the degradation of aggregated α-syn after uptake. However, the failure of intracellular protein degradation pathways may disturb their microenvironmental clearance function and contribute toward the processing and secretion of α-syn aggregates. α-syn can be degraded through two cellular processes: the ubiquitin-proteasome system (UPS) and the autophagy-lysosomal pathway (ALP).

### 3.1 Ubiquitin-proteasome system

**FIGURE 2:**
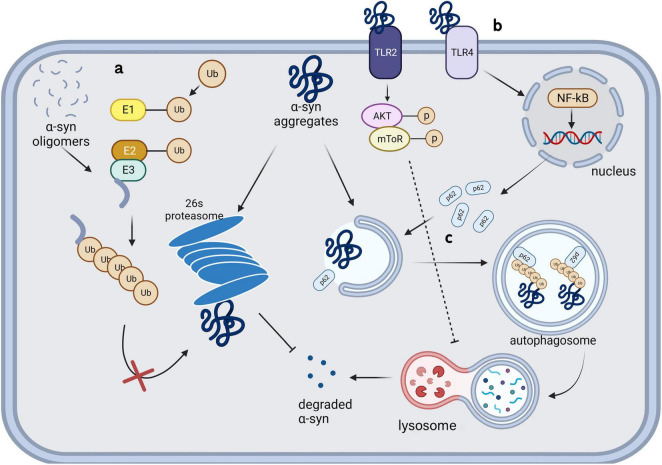
Intracellular clearance and degradation failure of α-synuclein in microglia.

UPS is an intracellular system that can specifically regulate the levels of cytosolic soluble proteins as well as damaged, misfolded, and mutant proteins (Pickart, 2001). The system comprises ubiquitin, ubiquitin-activating enzyme E1, ubiquitin-conjugating enzyme E2, ubiquitin ligase E3, deubiquitinating enzymes, and proteasomes. Proteasomes can recognize target proteins by adding polyubiquitin tags and degrade them (Figure 2a), which is the primary function through which monomeric and tetrameric α-syn are degraded under physiological conditions. Patients with PD have been shown to have impaired UPS function (McNaught and Jenner, 2001), suggesting its direct involvement in neurodegeneration. Although previous studies have shown that UPS is certainly involved in the processing of α-syn (Kumar et al., 2018; Emmanouilidou et al., 2010b; McKinnon et al., 2020), microglia, unlike neurons, are more inclined to clear α-syn via ALP. Knocking down of a deubiquitinating enzyme, Ubiquitin-specific protease 14 (USP14), in microglia decreases the phosphorylated α-syn in PFF treated BV-2 cells by increasing autophagy (Ding et al., 2024). In contrast, ALP deficiency critically damages α-syn clearance in microglia (Hong et al., 2024). These studies showed that the link between ALP and UPS allows ALP to compensate for the degradation failure of α-syn caused by UPS damage, but not vice versa. UPS may play a role in α-syn clearance in microglia, ALP is more crucial in this process.

### 3.2 Autophagy-lysosomal pathway

There has been significant interest in the role of ALP in PD. Autophagy is a self-digestion mechanism conducted by proteins encoded by highly conserved autophagy-related genes (ATGs). This process recycles organelles and provides energy under stressful conditions, including starvation; further, it is activated to allow clearance of damaged organelles, misfolded proteins, and exogenous pathogens. Paxinou et al. (2001) demonstrated that inhibiting lysosomes using ammonium chloride significantly increased the levels of α-syn aggregates in human embryonic kidney cells, suggesting the involvement of lysosomes in α-syn degradation. A subsequent genome-wide association study showed that mutations in leucine-rich repeat sequence kinase 2 (LRRK2), PTEN-induced kinase 1 (PINK1), Parkin RBR E3 ubiquitin-protein ligase (PARK2, Parkin), and Parkinson protein 7 (PARK7, DJ-1) increased the risk of PD; moreover, these genes are all related to ALP dysfunction (Pang et al., 2022; Narendra et al., 2010; Imberechts et al., 2022; Liu et al., 2017). Glucocerebrosidase, encoded by GBA1, catalyzes the hydrolysis of glucosylceramide into glucose and ceramide. Homozygous GBA1 mutations cause lysosomal storage disorders known as Gaucher disease, while heterozygous mutations have a minimal impact on lysosomal function and increase the risk of PD in carriers (Sidransky et al., 2009). In Klucken et al. (2012) used bafilomycin A1 to block the fusion of autophagosomes with lysosomes, which significantly increased α-syn toxicity in a PD model induced by C-terminally modified α-syn aggregates (Klucken et al., 2012). Subsequent studies confirmed that inhibiting lysosomal function led to an increase and decrease in the intracellular levels of monomeric and oligomeric α-syn, respectively, by up-regulating the extracellular secretion of high molecular aggregates. These differences in the effect may be attributed to the extracellular secretion of proteins with different conformations via different pathways (Poehler et al., 2014). Microglia have been proved to be the most effective cell type to degrade α-syn in the CNS *in vitro* (Lee H. et al., 2008). The exosomes secreted by α-syn transgenic neurons can damage the autophagy of microglia (Zhou et al., 2019). An *in vivo* experiment also confirmed that extracellular α-syn can significantly increase the accumulation of autophagy marker p62 in microglia (Tu et al., 2021).

#### 3.2.1 Heme-regulated kinase inhibitor

Heme-regulated kinase inhibitor (HRI), an eIF2α kinase, is widely expressed in various types of cells and responds to stress by phosphorylating elF2α. Mukherjee et al. (2021) used proteasome inhibitors to inhibit the UPS and found that HRI was involved in the degradation of α-syn aggregates by regulating B-cell lymphoma-2 associated athanogene 3 and heat shock protein 8.

#### 3.2.2 Toll-like receptor 4

Toll-like receptors 4 (TLR4), richly expressed on the surface of microglia, is confirmed to be crucial to α-syn clearance of microglia. TLR4 knockdown significantly reduce the α-syn phagocytosis by microglia and aggravate α-syn pathology (Venezia et al., 2021; Stefanova et al., 2011). Choi et al. (2020) found that microglia selectively uptake and degrade α-syn by autophagy through the TLR4-NF-κB-P62 pathway (Figure 2b). P62, as an inducible selective autophagy receptor, can self-oligomerize and bind to autophagosome membranes during neurodegenerative processes, and enhance the clearance of misfolded α-syn through directly binding and recruiting it into autophagosomes (Figure 2c; Choi et al., 2020; Tanji et al., 2015). TLR4 showed obvious configuration specificity for α-syn. Oligomers were identified as the most effective form as TLR4 agonists, compared with soluble and fibrillar forms (Hughes et al., 2019; Fellner et al., 2013). Conversely, this process can also be influenced by α-syn; specifically, the internalization of α-syn aggregates can damage microglial lysosomes and ultimately lead to autophagy inhibition or apoptosis through a series of pathways (Bussi et al., 2018). TLR4 and its downstream p38 and Akt-mTOR pathways can be affected by α-syn in the extracellular microenvironment, which leads to impaired autophagy in microglia. Subsequent animal studies have confirmed that microglial autophagic damage involving this pathway contributes to the pathogenesis underlying PD (Tu et al., 2021). Similar to α-syn present in the microenvironment, extracellular α-syn-containing exosomes can inhibit autophagy in BV-2 cells and increase α-syn aggregation in cells. This process may be facilitated by activation of the Akt-mTOR signaling pathway (Xia et al., 2019). Additionally, the function of TLR2 in degradation of internalized α-syn fibrils in microglia has been denied (Kim et al., 2021).

#### 3.2.3 Complement factor c1q

Mainly generated in microglia, complement factor c1q is involved in normal synapse pruning during development. A study showed that feeding PD mice exogenous α-ketoglutarate (AKG) can improve motor dysfunction, striatal aggregation of phosphorylated α-syn, and neuronal degeneration, which could involve increasing the level of docosahexaenoic acid (DHA) in the SN of mice. RNA-seq identification of genes affected by AKG and DHA suggested that both AKG and DHA alleviate α-syn pathology by promoting microglial autophagy, and this effect is abolished by knockdown of gene C1qa (Zhang et al., 2023).

#### 3.2.4 V-ATPase subunit G

V-ATPases are multi-subunit complexes situated on the lysosomal membrane, functioning as proton pumps to translocate protons into the lysosomal lumen. This process is crucial for maintaining a high acidic pH value within the lysosomal lumen. ATPase subunit G (V1G1) is one of the subunits of V-ATPases. Plasma exosomes containing α-syn can reduce the level of V1G1 in microglia and negatively alter the degradation of α-syn by impairing lysosomal function (Li et al., 2024).

In the past decade, multiple pathways related to the degradation of α-syn by microglia have been reported, which revealed a promising progress of PD that regulating ALP of microglia may improve the metabolism of α-syn. Nevertheless, it remains uncertain what directly triggers microgliosis and microglial degradation of neuronal α-syn instead of processing within neurons. Will it be a concentration threshold? Or certain interacting partners of α-syn is required? Answers to these questions may point a way to targets for clinical research.

## 4 Various α-syn conformations can be secreted by microglia in membrane-bound or free form

### 4.1 Unconventional protein secretion

Multiple studies have indicated that α-syn can be detected in cerebrospinal fluid (von Euler Chelpin et al., 2020), plasma/serum (Zubelzu et al., 2022; Wang et al., 2019), saliva (Shaheen et al., 2020), tears (Maass et al., 2020; Maass et al., 2020), and urine (Giri et al., 2022) of patients with PD, indicating its secretion to the extracellular space and diagnostic utility (Poggiolini et al., 2022). Moreover, α-syn can be released into the culture medium, *in vitro* (Sung et al., 2005). Since α-syn lacks the signal peptide sequence, it cannot be released to the extracellular space through the conventional endoplasmic reticulum (ER) targeting secretion pathway. Instead, intracellular α-syn aggregates exist in vesicles and are secreted to the extracellular space through a non-ER-dependent exocytosis process collectively termed as unconventional protein secretion (uCPSCPS) (Ejlerskov et al., 2013; Kim J. et al., 2018; Lee S. et al., 2010).

#### 4.1.1 Secretory autophagy

**FIGURE 3:**
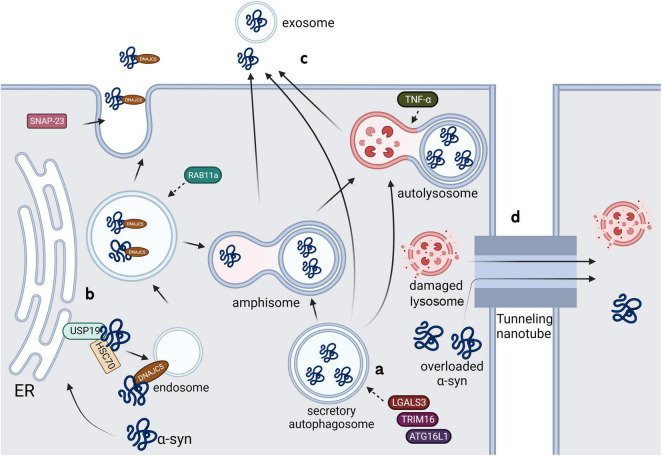
Possible pathways of α-syn secretion in microglia.

α-syn aggregates secrete through a special type of uCPSCPS known as secretory autophagy. Monomeric α-syn is secreted through a similar process; further, the secretion of both conformations is enhanced in the presence of lysosomal and mitochondrial dysfunction (Jang et al., 2010; Lee et al., 2013). LGALS3, a lectin, was found to be recruited to vesicles damaged by carrying cytoplasmic α-syn and to promote vesicle secretion through secretory autophagy in collaboration with autophagy-related proteins TRIM16 and ATG16L1 (Figure 3a; Burbidge et al., 2022). Rab11a, which is a member of the Rab family involved in membrane fusion, was initially considered to mediate the secretion of endosomal α-syn through exocytosis (Liu et al., 2009). However, a recent study showed that although Rab11 can regulate α-syn secretion, it does not involve the recycling endosomal pathway or multivesicular-exosomal pathway (Chutna et al., 2014); nonetheless, the specific mechanism remains unclear.

#### 4.1.2 Misfolding-associated protein secretion

The misfolding-associated protein secretion (MAPS) pathway is an intracellular protein quality control mechanism that transfers misfolded cytoplasmic proteins to the extracellular space in order to prevent cytotoxicity (Ye, 2018). In this process, an ER-associated deubiquitinase, USP19, can recruit misfolded proteins to the ER surface for deubiquitination. Subsequently, these proteins are encapsulated into late endosomes and secreted into the extracellular space (Lee et al., 2016). Usp19 is confirmed to be expressed in neurons, astrocytes and microglia, and a study showed that the loss of USP inhibits α-syn accumulation in neurons. Although researchers denied the role of α-syn transmission between neurons in this process by using primary neurons, the addition of microglia might yield totally different results (Schorova et al., 2023). The molecular chaperone, Hsc70, and its co-chaperone, DnaJC5, have been shown to directly interact with α-syn and increase its secretion through the SNAP23-mediated uCPSCPS (Fontaine et al., 2016), which suggests that this process may share a common plasma membrane system with MAPS. Another study showed that DnaJC5 can promote protein release mediated by USP19 and confirmed that USP19 acts upstream of DnaJC5 (Figure 3b; Xu et al., 2018).

#### 4.1.3 Exosomes

Studies have shown that α-syn-containing vesicles released to the extracellular space through calcium-dependent mechanism exhibit characteristics of exosomes (Emmanouilidou et al., 2010a). Exosomes are biologically active vesicles that are released into the extracellular environment and originate from multivesicular bodies (MVBs) or are created by directly budding at plasma membrane (Figure 3c; Pegtel and Gould, 2019). It has been found that α-syn oligomers can be released through exosomes, with this process being influenced by autophagic activity: blocking fusion of MVBs with lysosomes may increase α-syn levels in extracellular exosomes (Danzer et al., 2012). Another study showed that only 0.1–2% of secreted α-syn are present in vesicles, with the main location being the membrane of the extracellular vesicles (Gustafsson et al., 2018). These results suggested that, compared with lysosome-related pathways, exosome pathways may not be the main pathway for extracellular release of α-syn. Nonetheless, exosome-associated α-syn oligomers are more prone to uptake than free α-syn (Ye, 2018). It is confirmed that α-syn-containing exosomes released by microglia can be taken up by neurons and aggravate α-syn aggregation (Xia et al., 2019). Additionally, Guo et al. (2020) found that PFF upregulated the degradation of lysosomal structural protein LAMP2 by UPS through peli1, an E3 ubiquitin ligase, and therefore reduced the autophagic flux of microglia and increased the release of exosome-associated α-syn. Multiple factors regulate the release of α-syn through exosomes. Abe et al. (2024) found that LRRK2, a serine/threonine protein kinase, and its substrate Rab10 mediate the release of exosomal α-syn in microglia. PFF treatment can cause the accumulation of LRRK2 and Rab10 on lysosomal surface and induce the LRRK2 phosphorylation of Rab10 leading to extracellular release of exosomal α-syn (Abe et al., 2024). Similarly, Dai et al. (2023) reported that tumor necrosis factor receptor superfamily member 10B (TNFRSF10B) is essential in secretion of α-syn-containing exosomes from microglia, while the specific mechanism of it should be further explored. Additionally, knocking down Rab11a was found to prevent α-syn from entering MVBs and extracellular vesicles; contrastingly, this process promotes the overall secretion of α-syn through alternative pathways.

#### 4.1.4 Lysosomal exocytosis

First found in hematopoietic cells, lysosomal exocytosis is a Ca^2+^ regulated UCPs widely found in all types of cells, crucial to several cellular processes, including plasma membrane repair (Reddy et al., 2001), neurite outgrowth (Arantes and Andrews, 2006) and cargo secretion (Dou et al., 2012). Bae et al. (2022) found that neuronal α-syn can be released to the extracellular space through lysosomal exocytosis, which is regulated by microglial TNF-α, by knocking out the Rab27A gene, which controls the fusion of lysosomal and plasma membrane. Further research found that cJUN-N-terminal kinase (JNK) is pivotal in this process (Christensen et al., 2016). However, whether microglia release α-syn through lysosomal exocytosis and further affect the accumulation of α-syn in neurons still requires more evidence.

### 4.2 Tunneling nanotubes

First described in Rustom et al. (2004), tunneling nanotubes (TNTs) are elongated membrane channels constructed by F-actin and/or microtubules, which facilitate the transfer of ions, small molecules, nucleic acids, and large molecular substances such as proteins between directly adjacent cells (Gerdes et al., 2007). Several studies have confirmed that direct contact between neurons enhances the transfer of α-syn between cells (Abounit et al., 2016; Loria et al., 2017; Grudina et al., 2019). The propagation of α-syn through TNTs is regulated by the Wnt/Ca2+ pathway, which is a cellular cascade reaction that remodels the actin cytoskeleton (Vargas et al., 2019). Scheiblich et al. (2021) found that TNTs can transfer α-syn from overloaded microglia to functional neighbors, effectively degrading it and increasing the microglial viability (Figure 3d), and lately confirmed that neurons can transfer pathological α-syn into neighbor microglia through TNTs as well (Scheiblich et al., 2024). This transfer not only occurs between neurons and microglia, but also between astrocytes and microglia, where TNTs can directly deliver α-syn to adjacent microglia for degradation (Rostami et al., 2021). TNTs are crucially involved in the intercellular transfer of damaged α-syn- carrying lysosomes (Dilsizoglu Senol et al., 2021), and microglia are also found to transport mitochondria to α-syn-containing neurons through TNTs (Chakraborty et al., 2023), which may contribute to the degradation of cytotoxic aggregates. The release of α-syn under overload demonstrates the limit of protective effect and potential pathogenic ability of microglia in PD. Given the high toxicity α-syn derived from microglia to neurons, identifying what upregulates the release of exosomal α-syn and screening effective intervention targets may become a promising direction.

## 5 Discussion

Propagation of pathologic α-syn is a prominent feature of PD, with previous studies mainly focusing on neurons. However, increasing evidence suggests that microglia are closely associated with the onset and progression of α-synucleinopathy. The traditional view believed that microglia internalize and degrade pathological α-syn from cell debris after the death of neurons. However, recent studies have shown that microglia are crucially involved in the internalization, processing and spread of α-syn in earlier stages of PD. Microglia can obtain α-syn from functional neurons and may prevent neurodegeneration, or conversely process α-syn into a more neurotoxic form under excessive burden of α-syn. This also raises a key question: how effective will our intervention targeting the pathways mentioned above be? Some potentially promising approaches, such as autophagy enhancement through α-syn/TLRs/NF-κB pathway or exosome inhibition, still require further investigation. Currently, there is a lack of adequate pharmacal research targeting the pathological mechanisms of microglia in PD. Additionally, certain neuroimmune markers during the early stages of disease, when microglia remain normal homeostatic functions, may be detectable, and alteration of these markers may assist early diagnosis. There is major interest in α-syn as among the main focuses of disease-modifying therapies for PD, and the dysregulation of microglial functions in PD may be a decisive target for clinical intervention.
